# miR-22 Forms a Regulatory Loop in PTEN/AKT Pathway and Modulates Signaling Kinetics

**DOI:** 10.1371/journal.pone.0010859

**Published:** 2010-05-27

**Authors:** Nadav Bar, Rivka Dikstein

**Affiliations:** Department of Biological Chemistry, The Weizmann Institute of Science, Rehovot, Israel; University of São Paulo, Brazil

## Abstract

**Background:**

The tumor suppressor PTEN (phosphatase and tensin homolog) is a lipid phosphatase that converts PIP3 into PIP2 and downregulates the kinase AKT and its proliferative and anti-apoptotic activities. The FoxO transcription factors are PTEN downstream effectors whose activity is negatively regulated by AKT-mediated phosphorylation. PTEN activity is frequently lost in many types of cancer, leading to increased cell survival and cell cycle progression.

**Principal Findings:**

Here we characterize the widely expressed miR-22 and report that miR-22 is a novel regulatory molecule in the PTEN/AKT pathway. miR-22 downregulates PTEN levels acting directly through a specific site on PTEN 3′UTR. Interestingly, miR-22 itself is upregulated by AKT, suggesting that miR-22 forms a feed-forward circuit in this pathway. Time-resolved live imaging of AKT-dependent FoxO1 phosphorylation revealed that miR-22 accelerated AKT activity upon growth factor stimulation, and attenuated its down regulation by serum withdrawal.

**Conclusions:**

Our results suggest that miR-22 acts to fine-tune the dynamics of PTEN/AKT/FoxO1 pathway.

## Introduction

The tumor suppressor phosphatase and tensin homolog known as PTEN is a lipid phosphatase involved in the regulation of the cell cycle. Inactivating mutations of this gene or of its regulators contribute to the development of certain cancers [1], [2]. The main substrates of PTEN are phosphoinositides, particularly phosphatidylinositol-3,4,5-triphosphate (PIP3) whose intracellular levels are reduced following its dephosphorylation by PTEN to a diphosphate product (PIP2), and consequently AKT kinase activity and signaling are restrained. The FoxO family of transcription factors is one of the AKT downstream direct substrates. FoxO proteins are phosphorylated by AKT, and translocated from the nucleus to the cytoplasm where they are degraded via the ubiquitin–proteasome pathway [3]. When PTEN is active and AKT activity is suppressed, FoxO proteins are able to enter the nucleus and upregulate genes that promote cell cycle arrest or apoptosis [3]. The PTEN mRNA has an unusually long 3′UTR, about 3.3 kb, suggestive of tight post-transcriptional control of PTEN expression. Within the PTEN 3′UTR are several conserved microRNA target sites and PTEN has been shown to be repressed by miR-21, 214, 216a, 217, 17–92 and 26a [4], [5], [6], [7], [8].

MicroRNAs are small 19–24 bp long non-coding RNAs which function primarily to down-regulate gene expression in all metazoan eukaryotes. The microRNAs specifically interact with target mRNAs through base pairing preventing their translation (for recent review see [9], [10] and sometimes promoting their degradation [11]. A single microRNA can regulate a large number of target mRNAs [12], and on the other hand, a single gene may be regulated by more then one microRNA as is the case with PTEN. Since the human genome encodes for several hundred different microRNAs, they can be expected to regulate thousands of genes [13]. MicroRNAs are encoded by specific genes or are processed (via the enzyme Dicer) from a variety of different RNA sources, including introns, 3′ UTRs of mRNAs and long noncoding RNAs [9], [14]. Most microRNAs are transcribed by RNA polymerase II (Pol II) [15], [16], though several have been shown to be transcribed by RNA polymerase III (Pol III) [17], [18].

The most important requirement for microRNA base pairing with an mRNA target in animal cells is a continuous and perfect complementarity of microRNA nucleotides 2–8, known as the seed region, to the 3′ UTR of the target mRNA [19]. Several computational programs have been generated to predict microRNAs targets [20], [21], [22], most of them utilizing the complementarity between the microRNA seed and the 3′UTR of the target mRNA and conservation of the site between species. However, of the large number of predicted targets for each microRNA a significant fraction is false, indicating there are also other parameters, yet to be discovered, that determine microRNA-target association.

Some miRNAs are expressed at higher levels than their targets (i.e. cell type specific or developmentally regulated miRNA), while other miRNAs are co-expressed together with their target in the same cell. Ubiquitously expressed miRNAs belong to the latter class, and it was suggested that their function is to fine-tune target expression at a post-transcriptional level [23], [24]. MiR-22 is one of very few ubiquitously expressed microRNAs [25], and is therefore likely to be involved in buffering cellular activities that are common to all cells. In the present study we characterized miR-22 function and regulation. Our findings suggest that miR-22 controls the signaling kinetics of PTEN/AKT/FoxO1 pathway and is by itself target for regulation by this pathway.

## Results

### miR-22 is transcribed by RNA polymerase II and has a strong TATA-less promoter

**Figure 1:**
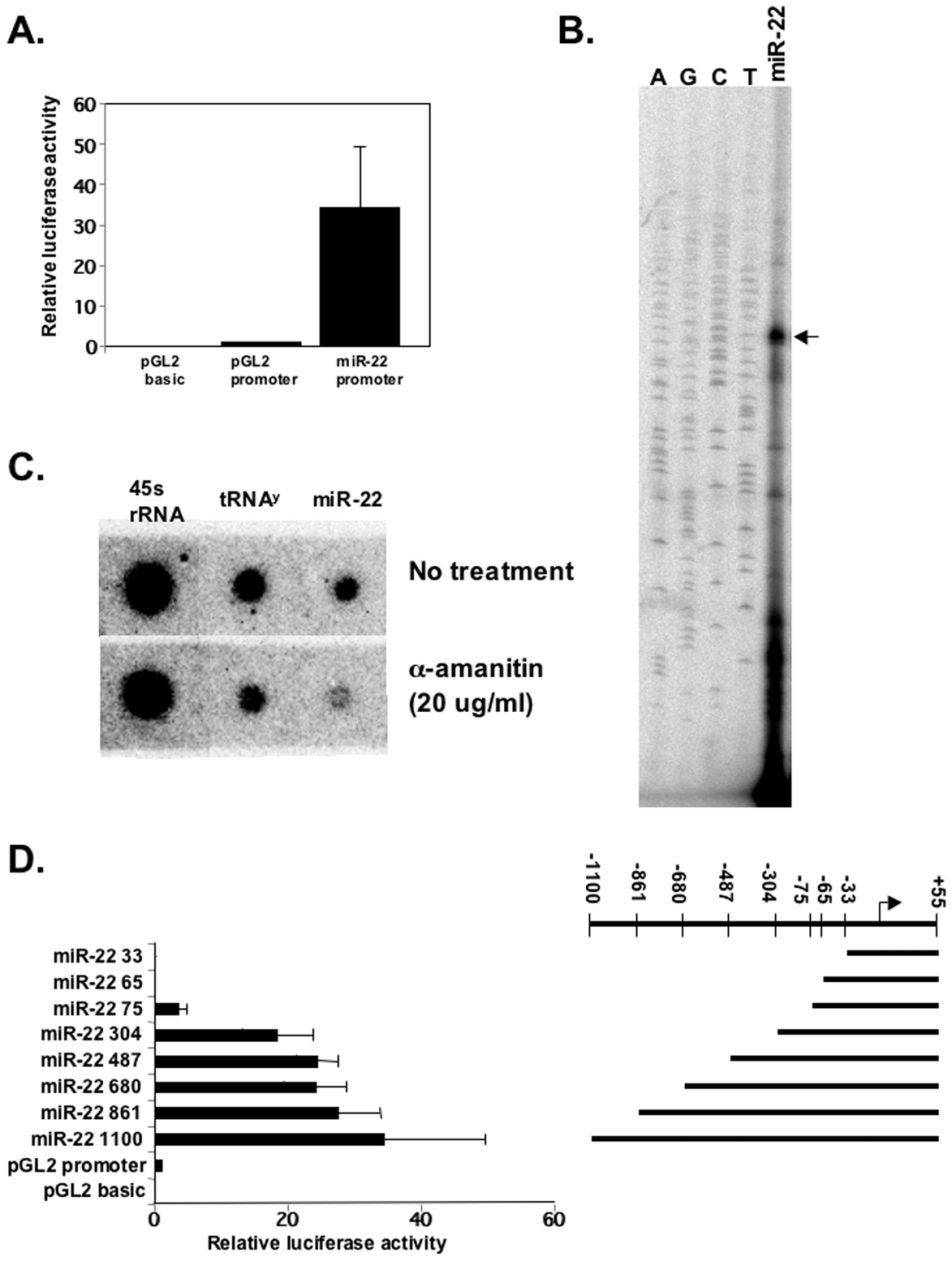
Identification of miR-22 promoter. **A.** A 1155 bp fragment of miR-22 predicted promoter from −1100 to +55 was cloned upstream to the luciferase gene in the promoter-less pGL2 plasmid and transfected into HEK 293T together with RSV-renilla that served as internal control for transfection efficiency. pGL2-basic and pGL2-SV40-promoter were also transfected and served as negative and positive controls respectively. 24 hours later the relative luciferase activities were determined. The results represent the mean +/− SD of 7 independent transfection experiments. **B.** Determination of miR-22 TSS. The luciferase reporter gene under the control of miR-22 promoter was transfected into HEK293T cells and 24 hours after transfection RNA was extracted and analyzed by primer extension using a primer complementary to the 5′-end of the luciferase gene. The labeled miR-22 derived cDNA was run on a denaturing 8% urea-polyacrylamid gel alongside a sequencing reaction marked by A, G, C and T. **C.** Analysis of sensitivity of endogenous miR-22 transcription to α-amanitin. Nuclei were isolated from HEK293T cells and subjected to a run-on assay in the absence or presence of 20 µg/ml α-amanitin using ^32^P-labeled UTP. Labeled RNAs were isolated and then hybridized to membranes that were dot-blotted with RNA pol I gene, 45s rRNA; RNA pol III gene tRNA^Y^ and miR-22 transcripts as indicated. **D.** Analysis of miR-22 promoter organization. The miR-22 promoter was dissected from the 5′ as shown schematically and luciferase activity of the different constructs was determined as described in A. The results represent the mean +/− SD of 4–6 independent transfection experiments.

miR-22 is a widely expressed microRNA [25] whose regulation and target genes have so far been poorly investigated. As a first step in its characterization we set out to identify the miR-22 promoter. We retrieved the pre-miR-22 sequence and through a Blast search identified a 1.3 kb full length cDNA (DKFZp686O06159) containing the putative most 5′-end. This cDNA does not seem to encode for a protein. We then cloned, by genomic DNA PCR, a DNA fragment bearing the predicted promoter from −1100 to +55, upstream to a promoter-less luciferase reporter gene. The plasmid was transfected into HEK293T cells and luciferase activity was measured and compared to the activities of the promoter-less parental plasmid or the SV40 early promoter that was used as a positive control. The putative promoter of miR-22 displayed very strong activity with a luciferase activity approximately 15 fold higher than that of the SV40 promoter (Fig 1A). We next determined the position of the transcription start site (TSS) of the mRNA produced by the reporter gene using primer extension with a ^32^P-labeled primer complementary to the luciferase gene. Fig. 1B shows that the miR-22 promoter directs a major TSS located exactly at the position of the most 5′ end of the full length cDNA.

The miR-22 promoter lacks TATA box and Initiator core promoter elements, but nevertheless directs a single major TSS which is unusual in TATA-less promoters. We therefore tested whether miR-22 is transcribed by RNA polymerase II (pol II). We performed a nuclear run-on assay using the low concentrations (20 µg/ml) of α-amanitin that inhibit pol II but not pol I and III. Isolated nuclei from HEK293T cells were incubated with NTPs which included the labeled ^32^P-UTP. Labeled RNAs were then isolated and hybridized to membranes blotted with 45S rRNA (Pol I), tRNA^tyr^ (Pol III) and primary miR-22. The results show that while 45S rRNA and tRNA^tyr^ transcription was not significantly affected by 20 µg/ml of α-amanitin, transcription of miR-22 was clearly diminished (Fig. 1C). The involvement of Pol II in miR-22 transcription was verified by chromatin immunoprecipitation assay (ChIP) with Pol II antibodies (Fig. S1).

We next determined which DNA elements in the promoter are important for regulating miR-22 transcription. We constructed a series of 5′ deletions of the miR-22 promoter (Fig. 1D, right panel) and analyzed their activity in HEK293T cells (Fig 1D, left panel). No significant differences were observed between the whole promoter, up to -1100, and 5′ deletion up to position −487 relative to the TSS. Deleting the region between −487 to −304 slightly decreased promoter activity whereas deleting the region between −304 to −75 substantially decreased promoter activity indicating that an important regulatory element(s) lies in this region. Further deletion of 10 bp between −75 to −65 caused complete loss of transcriptional activity. Thus the region between −75 to −65 contains as an essential regulatory element. Analysis of this element in several programs for transcription factor binding sites revealed a lack of perfect match with the consensus of any known transcription factor, though it bears significant resemblance to the NRF-1 binding site consensus. However neither overexpression of NRF-1 nor expression of a dominant negative mutant nor NRF-1 RNAi significantly affected miR-22 promoter activity (Fig. S2), suggesting that a transcription factor other than NRF-1 activates the miR-22 promoter through this element.

### miR-22 downregulates PTEN expression

**Figure 2:**
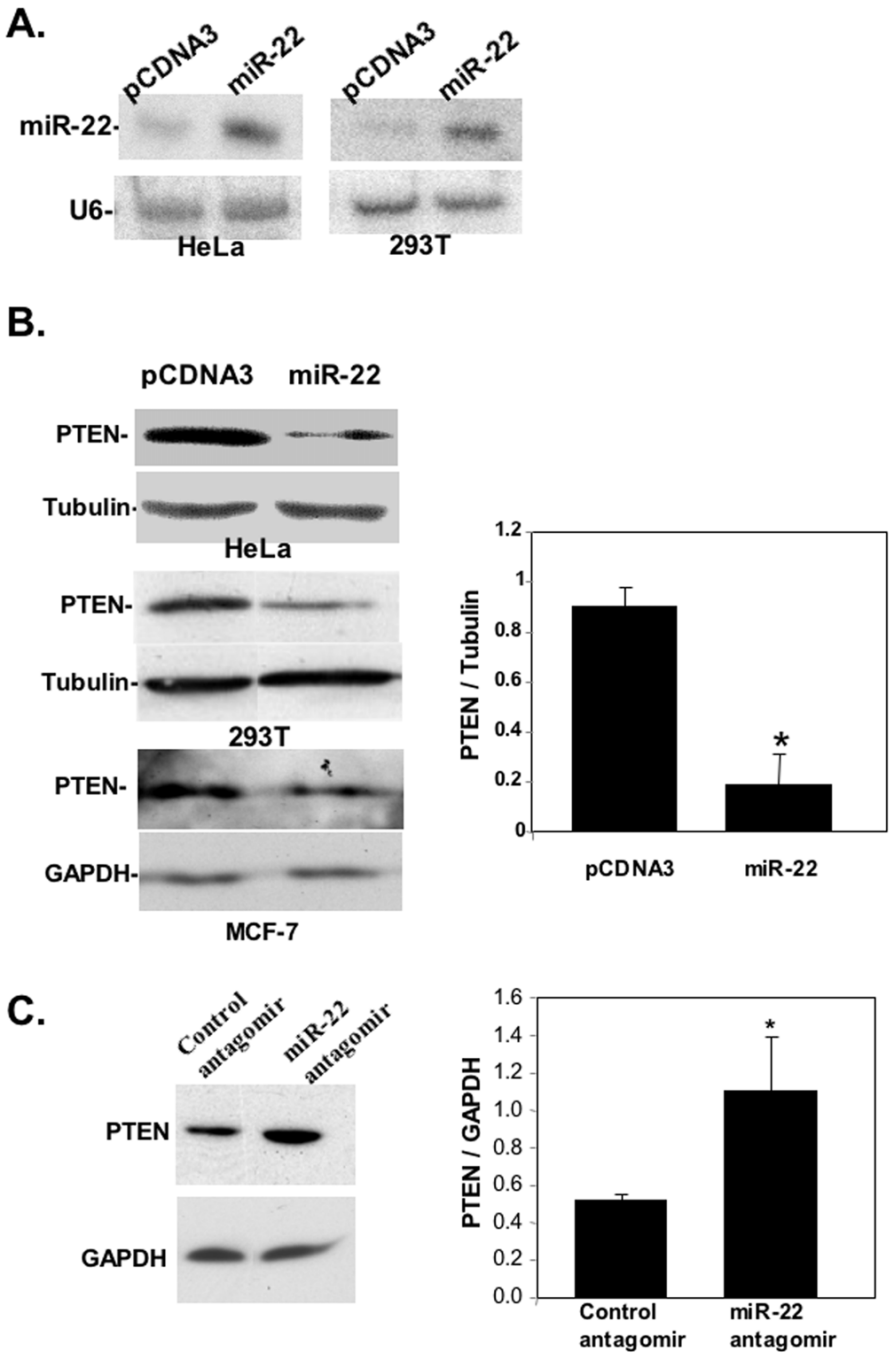
PTEN is a direct target of miR-22. **A.** Northern blot analysis with miR-22 and U6 probes of small RNAs extracted from HeLa or HEK293T cells, as indicated, transfected with miR-22 or parental (pCDNA3) expression plasmids. **B.** Left panel shows western blot analyses, with PTEN, tubulin and GAPDH antibodies, of cell lystates prepared from HeLa, HEK293T and MCF-7 cells, as indicated, transfected with miR-22 expression plasmid or the parental vector pCDNA3. Quantification, by densitometry, of 3 independent transfection experiments in HeLa cells is shown in the right panel in which * indicates p<0.01. **C.** Representative western blot analysis with PTEN and GAPDH antibodies of cell lystates prepared from HeLa cells transfected with miR-22 or control antagomir expression plasmids. Quantification of PTEN levels of 4 independent transfection experiments is shown in the right panel in which * indicates p<0.05.

To identify potential targets of miR-22 we constructed a miR-22 expression plasmid and confirmed, by northern blot, that it directs expression of mir-22 RNA (Fig. 2A). Using the TargetScan algorithm [20] we selected four high scoring predicted target genes PTEN, SIRT1, SP1 and p300 to examine whether miR-22 affects their expression. The miR-22 expression plasmid was transfected into HeLa cells and the selected proteins were determined by western-blot. PTEN is downregulated by miR-22 expression in HeLa cells (Fig. 2B left panel, top) but the levels of the other proteins were not significantly changed (Fig. S3). Likewise, in HEK293T and MCF-7 cells, expression of miR-22 reduced PTEN protein levels. Quantification of several transfection experiments in HeLa cells shows that PTEN downregulation is highly significant (Fig. 2B right panel). As expected reduction of PTEN levels by miR-22 enhanced the phosphorylated active form of AKT (Fig. S4).

To determine whether endogenous PTEN is targeted for regulation by endogenous mir-22 we constructed a plasmid expressing miR-22 antagomir, a small RNA that can forms a duplex with the endogenous miR-22 and neutralizes it. HeLa cells were transfected with miR-22 antagomir or with a control vector expressing a similar size non-relevant sequence. Western-blot analysis showed a significant increase in PTEN protein level upon inactivation of endogenous miR-22 (Fig. 2C), providing evidence that PTEN is targeted for regulation by the endogenous miR-22.

### miR-22 targets PTEN through a conserved site at the 3′UTR

**Figure 3:**
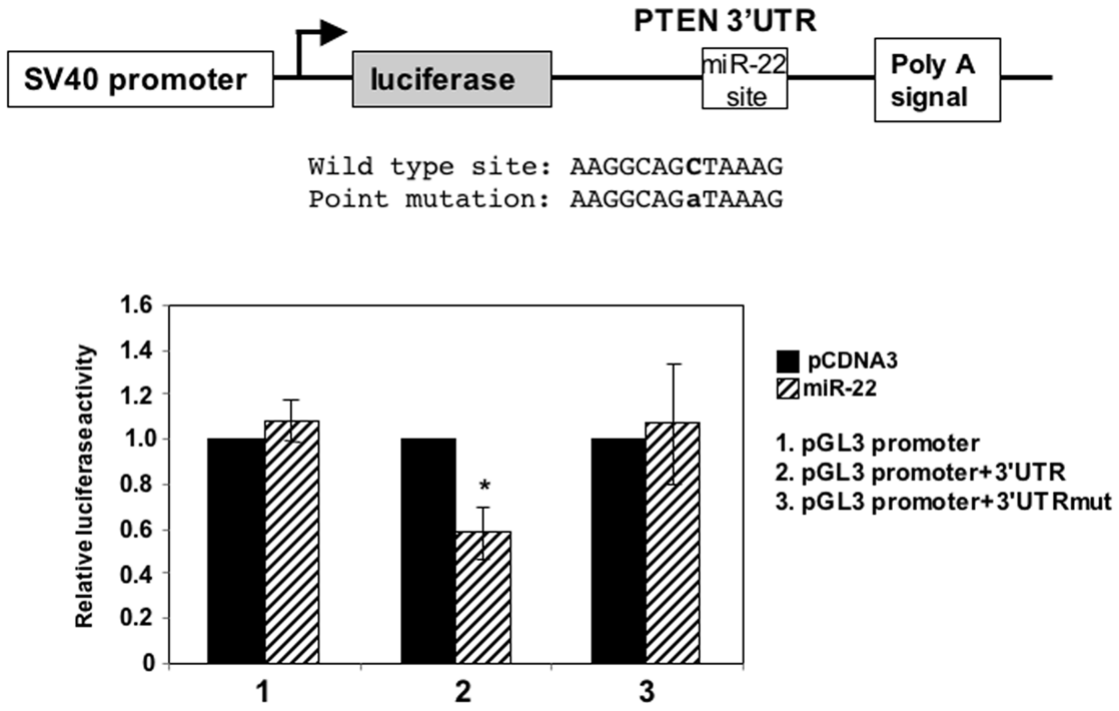
miR-22 targets PTEN through a site at the 3′UTR. A schematic representation of a reporter gene used to analyze the miR-22 target site of PTEN 3′UTR is shown in the upper panel. The sequences of the wild type and mutated target site are shown. The wild type and mutated reporter plasmids were co-transfected into HEK293T cells together with miR-22 expression plasmid or the parental vector pCDNA3 and RSV-renilla that served as internal control. The activity of each construct without miR-22 was set to 1. The results represent the average +/− SD of four independent transfection experiments in which the bar marked with * indicates p<0.01.

miR-22 was predicted to target PTEN through a single conserved site at the 3′UTR of PTEN mRNA. To examine this possibility a fragment of the PTEN 3′UTR, containing the predicted target site, was cloned downstream to the luciferase gene in the pGL3-SV40-promoter plasmid. As a control a single point mutation was introduced in the seed of the target sequence (Fig. 3). Co-transfection of miR-22 or parental (pCDNA3) expression plasmids with the wild type and mutant reporter genes decreased the luciferase activity of the reporter bearing the wild type but not the mutated PTEN 3′UTR. Likewise miR-22 expression did not affect the parental reporter gene (Fig. 3). These results support the idea that miR-22 exerts its effect on PTEN by interacting directly with a target site on the 3′UTR.

### miR-22 expression influences PTEN/AKT signaling kinetics

**Figure 4:**
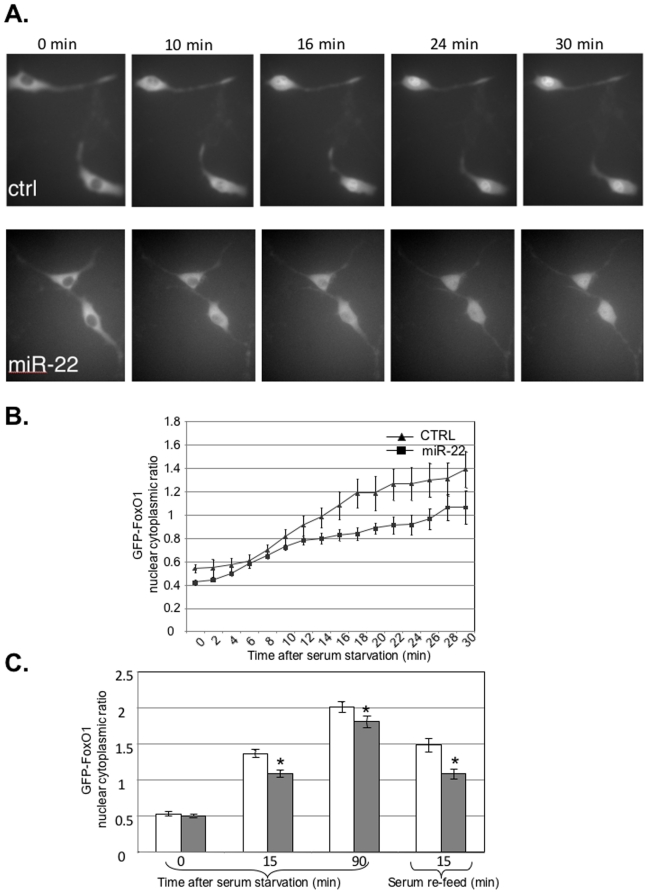
The effect of miR-22 expression on the sub-cellular localization of FoxO1. **A.** NIH3T3 cells were co-transfected with FoxO1-GFP together with either miR-22 expression plasmid or the parental pCDNA3. 48 hours after transfection FoxO1-GFP from 10 fields of each transfection was visualized by Time-Lapse microscopy before and after serum deprivation. Representative images from the indicated time points after serum withdrawal are shown. **B**. Quantitative analysis of changes in FoxO1-GFP localization after serum starvation at 2 minutes intervals up to 30 minutes. The measurement was carried out by densitometry of the fluorescent signal in the cytoplasm and the nucleus of each cell. The data is presented as the ratio between nucleus and cytoplasm. **C**. Analysis of changes in FoxO1-GFP subcellular localization as in B using fluorescent microscope. Images of 15–20 fields (30–50 cells) of each transfection were taken at four different time points: before starvation (time 0), 15 and 90 minutes after replacing the cell medium to serum-free medium, and 15 minutes after addition of serum to the sarved cells (Re-Feed). The results are the sum of three independent biological repeats. The asterisks indicate that the differences between control and miR-22 in 15, 90 minutes after starvation and reefed points are statistically significant (p = 8×10^−5^, 3×10^−2^, and 2×10^−4^ respectively).

To find the consequences of interference of PTEN expression by miR-22 we measured AKT activity in response to growth factor withdrawal and stimulation. To probe the endogenous AKT activity in living cells we followed the sub-cellular localization of FoxO1, an established direct target of this kinase [3]. Phosphorylation of FoxO1 by AKT results in its accumulation in the cytoplasm, while inactivation of AKT causes FoxO1 translocation into the nucleus. NIH3T3 cells were co-transfected with FoxO1-GFP together with either miR-22 expression plasmid or the parental pCDNA3 and 48 hours after transfection FoxO1-GFP localization was monitored by fluorescent microscopy. Under conditions in which cells are continuously grown in the presence of serum, FoxO1-GFP is localized in the cytoplasm of most cells both in control and in miR-22 expressing cells (see Fig. 4C, time 0). Therefore we determined the effect of miR-22 expression on the dynamics of AKT signaling by following the changes in FoxO1 localization after serum withdrawal in live cells using Time-Lapse microscopy. Images were taken every two minutes up to 30 minutes after starvation. Fig. 4A and B show that in control cells FoxO1 starts accumulating in the nucleus within 10 minutes after growth factor starvation and continues to accumulate during the course of the experiment. Interestingly, miR-22 expression causes a delay in the nuclear accumulation of FoxO1, consistent with higher basal AKT activity as a consequence of PTEN down regulation by miR-22. To verify this observation we scaled up the analysis and used a standard fluorescent microscope that can handle a larger number of cells. Images of randomly chosen 30-50 cells were taken at four different time points: before starvation (time zero), 15 and 90 minutes after serum starvation, and 15 minutes after serum addition to the starved cells (re-feed). Densitometric measurements of images from 3 independent biological replicates (Fig. 4C) show that under continuous growth with serum, there is no difference between control and miR-22 expressing cells (time 0). In miR-22 expressing cells, however, there is significant attenuation of nuclear accumulation of FoxO1 at the early time point (15 min) after serum starvation, as we observed with time-lapse microscopy. This effect is less pronounced at 90 minutes. Replenishing the starved cells with growth factors in order to induce AKT activity resulted in the rapid nuclear exit of FoxO1 (re-feed columns), and this effect is accelerated in cells expressing miR-22, which again is indicative of higher levels of active endogenous AKT. To validate that the translocation of FoxO1 from the nucleus to the cytoplasm is AKT dependent we co-transfected into NIH3T3 cells miR-22 with a dominant negative mutant of AKT. Inhibition of AKT activity caused nuclear retention of FoxO1-GFP at all times and in the absence or presence of either growth factors or miR-22 (Fig. S5).

### miR-22 expression is modulated by PTEN/AKT/FoxO1 pathway

**Figure 5:**
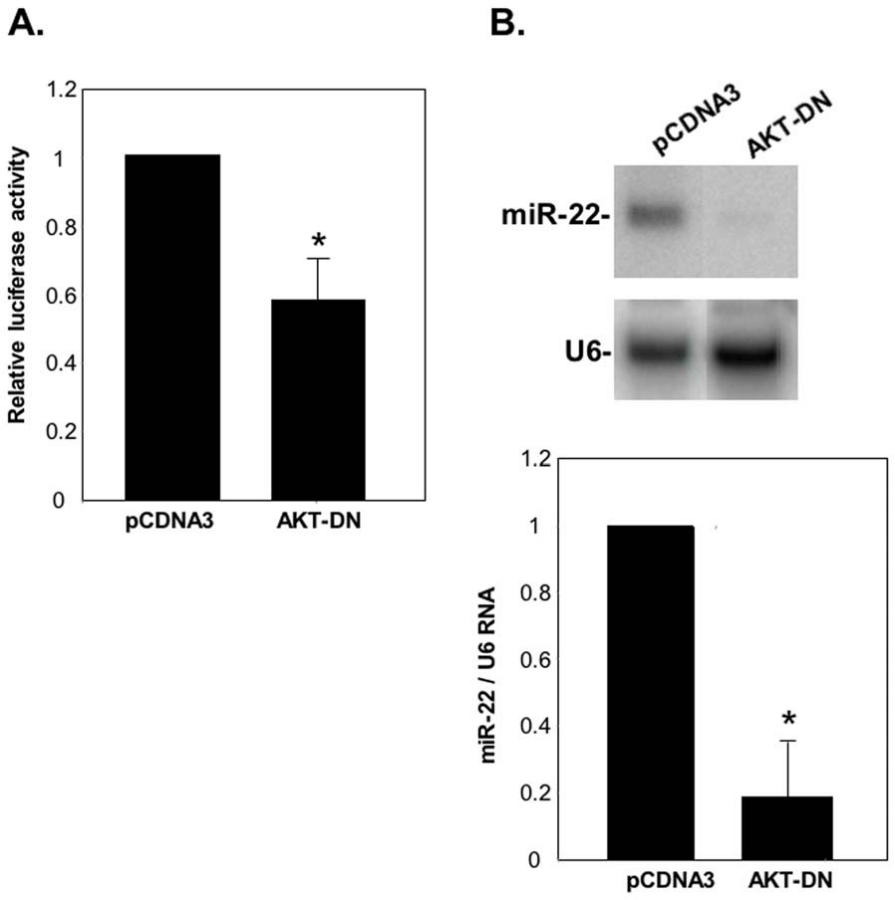
The PI3K/AKT pathway affects miR-22 expression. **A.** The luciferase reporter gene under the control of miR-22 promoter was co-transfected into HEK293T cells together with either an empty expression plasmid (pCNDA3) or plasmid expressing a dominant negative mutant of AKT (AKT-DN). 24 hours after transfection cells were harvested and the relative luciferase activity was determined. The results represent the mean +/− SD of three independent experiments. The asterisk indicates statistically significant difference (p<0.01). **B.** HeLa cells were transfected with either dominant negative AKT or empty expression plasmid together with puromycin resistant gene plasmid. 24–48 hours after transfection small RNAs were extracted and subjected to Northern blot with miR-22 and U6 probes as indicated. A representative Northern blot is shown in the top panel and densitometric quantification of three independent transfection experiments is shown in the bottom panel. The asterisk indicates statistically significant difference (p<0.001).

We next asked whether the PTEN/PI3K/AKT pathway itself could influence miR-22 levels. HEK293T cells were transfected with the miR-22 promoter luciferase reporter gene together with an expression plasmid of a dominant negative mutant of AKT, an inhibitor of AKT. The results revealed a significant decrease in luciferease activity by dominant negative AKT mutant (Fig 5A) suggesting that the AKT signaling pathway enhances miR-22 expression at the transcriptional level. To test this possibility directly, we examine the effect of AKT inhibition on the endogenous levels of miR-22 RNA. HeLa cells transfected with a dominant negative mutant of AKT and 24–48 hours after transfection the levels of miR-22 were analyzed by northern blot. Fig. 5B shows substantially lower levels of endogenous miR-22 in cells expressing the AKT inhibitor, confirming that AKT enhances miR-22 expression.

### miR-22 and PTEN share an important regulatory element in their promoters

**Figure 6:**
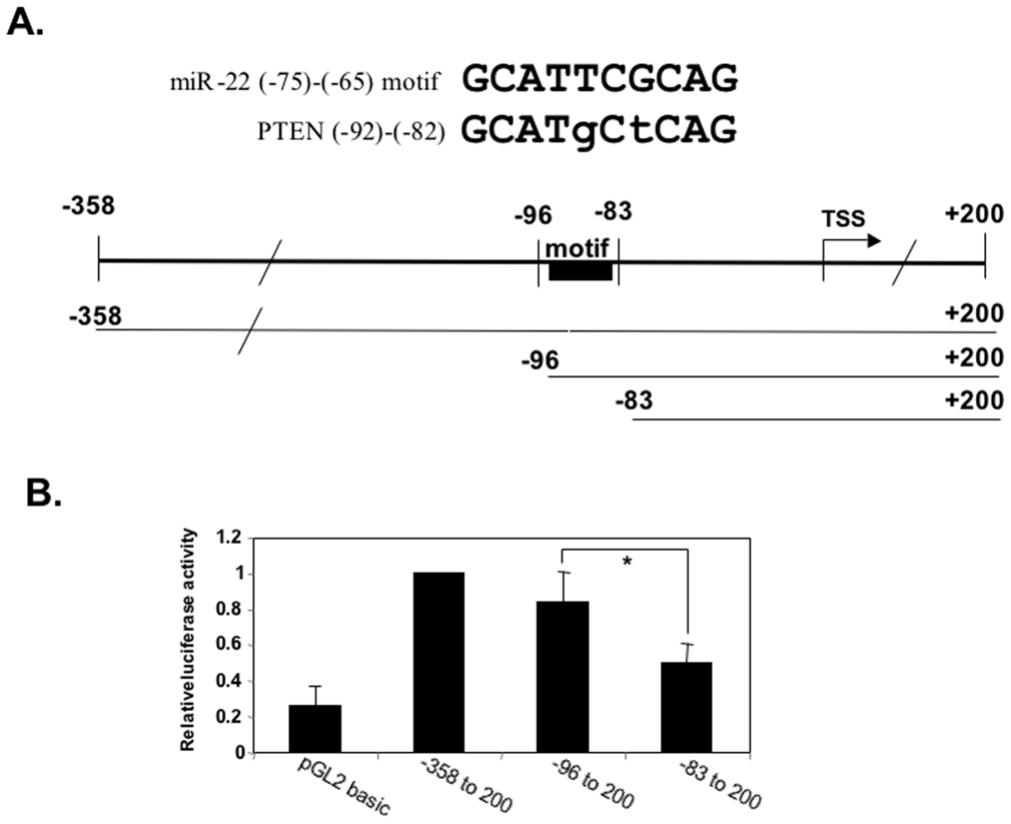
Identification of an important transcription regulatory element in PTEN promoter that is similar to the −75 to −65 motif of miR-22 promoter. The upper panel shows sequence alignment between the −75 to −65 motif of miR-22 promoter and the −92 to −82 region of PTEN promoter. Fragments of PTEN promoter with progressive 5′ deletions that are schematically shown, were cloned upstream to a luciferase gene in the promoter-less pGL2 basic and then transfected into HEK293T cells together with RSV-renilla that served as internal control for transfection efficiency. 24 hours later the relative luciferase activities were determined. The results represent the mean +/− SD of 7 independent transfection experiments. The bars marked with asterisk indicate statistically significant difference p<0.001.

To understand why PTEN but not the other predicted targets that we examined was affected by miR-22, we raised the possibility that a true target may have additional features in common with the microRNA, such as transcriptional control. To examine this possibility we analyzed the sequence of the previously characterized PTEN promoter [26] and found that it has a sequence closely similar to the essential −75 to −65 element of the miR-22 promoter. This sequence is also located proximal to the TSS (−92 to −82) as in miR-22 (Fig. 6, upper panel) and it is absent from the promoters of the false targets p300, SIRT1 and Sp1. To examine whether this sequence is important for PTEN promoter activity, the PTEN proximal promoter from −358 to +200 was cloned in front of a luciferase reporter gene. Subsequently we constructed two 5′ deletion mutants, one up to position −96 which contains the common sequence element and the second 13 bp shorter, without it. These constructs were transfected into HEK293T cells and luciferase activity was measured. As shown in Fig. 6 the −385 to +200 displayed a significant promoter activity compared to the promoter-less pGL2 basic construct. Most of PTEN promoter activity is retained upon deletion to -96 position whereas a significant reduction in activity is observed when the common sequence element is deletedas in the −83 mutant. These results indicate that the common sequence element is important for PTEN promoter activity as it is for miR-22 promoter activity. They also support the idea that genuine targets of microRNAs share common regulatory characteristics with the microRNAs in addition to the binding site on the target 3′UTR.

## Discussion

**Figure 7:**
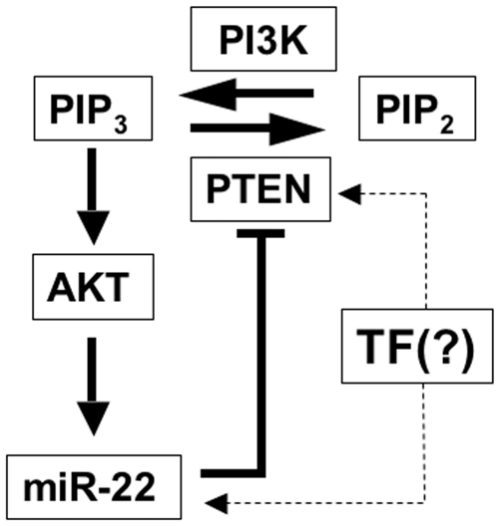
A model showing PTEN/PI3K/AKT pathway with miR-22 and the regulatory loop exerted by miR-22.

One of the most important signaling cascades that modulate cell cycle progression and cell survival in many cell types is PTEN/PI3K/AKT which is deregulated in several different kinds cancers [1], [2]. In the present study we have identified and characterized a previously unknown constituent of this pathway, miR-22. miR-22 targets PTEN directly through a conserved site on the PTEN 3′UTR. The PTEN/AKT pathway is functional in most cell types and tissues and so is the expression of miR-22. Therefore miR-22 does not serve to eliminate PTEN expression, but rather, to fine-tune its expression levels according to the physiological needs of the cell. This idea is consistent with our finding that miR-22 acts to modify the kinetics of the PTEN/AKT/FoxO1 signaling upon changes in extracellular signals, but has little effect on this signaling pathway under steady state conditions. Interestingly, expression of miR-22 itself is affected by the PTEN/AKT pathway, in a way that generates a feed-forward regulatory loop (Fig. 7). In this regulatory circuit miR-22 suppresses PTEN expression leading to enhancement of AKT activity, which in turn upregulates miR-22 transcription (Fig. 7). Thus, in this regulatory network, miR-22 acts to enhance AKT signaling (Fig. 7). Time-resolved live cell imaging of AKT activity revealed that this circuit serves to accelerate the signaling through this pathway.

Accumulating evidence reveals that a microRNA can regulate multiple targets and one gene can be under the regulation of several microRNAs [12]. Accordingly, both miR-22 and PTEN were shown to be associated with other regulatory systems. PTEN was recently found to be regulated by multiple miRs including miR-21 miR-214, 216a, 217, 17–92 and 26a [4], [5], [6], [7], [8]. Considering the central role that PTEN has in cell cycle progression and survival and its unusually long 3′UTR PTEN levels are probably influenced by additional microRNAs, some of which would exert their effect in a cell type specific manner compatible with the site of their expression. A recent study reported that miR-22 directly suppresses the expression of the estrogen receptor alpha (ERα) in breast cancer cells [27]. While regulation of PTEN by miR-22 is likely to occur in many different cell types, regulation of ERα will take place only in those tissues that express the ERα receptor.

Prediction of microRNA targets is based upon the complementarity between the seed of the microRNA and a sequence in the 3′UTR of the mRNA and its conservation among species [19], [20]. However it appears that in spite of the presence of these features, many of the predicted targets are refractory to microRNA regulation. Of the four miR-22 target candidates we selected, PTEN, p300, SIRT1 and Sp1, only PTEN was sensitive to miR-22. To understand what additional features characterize a true microRNA target we examined their transcription regulatory parameters. Our findings revealed that in addition to the presence of a miR-22 target site in the 3′UTR of PTEN, miR-22 and PTEN, but not the refractory predicted targets, share a common transcription regulatory element in their promoters. Considering the regulation of miR-22 by AKT and intriguing possibility is that PTEN is also affected by AKT at the transcriptional level. Thus it may be useful to look for common regulatory features between a microRNA and its target to gain a better estimation of their potential to interact.

In conclusion, we have revealed mutual regulation between the tumor suppressor PTEN and the microRNA miR-22. PTEN was identified as a direct target for negative regulation by miR-22, and the PTEN downstream target AKT activates miR-22 transcription. In the PTEN/AKT signaling pathway the major function of miR-22 is to modulate pathway dynamics in response to extracellular signals.

## Materials and Methods

### Plasmids construction

The promoters of miR-22 and PTEN were amplified by genomic PCR and cloned into the promoter-less pGL2-basic (Promega) via SmaI and HindIII sites. Dissections of miR-22 and PTEN promoters were similarly constructed, using the same reverse primer as for the parental construct. To construct the reporter gene with the PTEN 3′UTR, we used the pGL3-promoter (Promega) in which luciferase is under the control of the SV40 early promoter. First, a multiple cloning site was inserted downstream to the luciferase gene at the XbaI site. Then a PTEN 3′UTR fragment (from 2910 to 2960) was amplified from genomic DNA and cloned downstream to the luciferase gene via SacII and EcoRI sites. To obtain the PTEN 3′UTR point mutation, oligonucleotides bearing the designed sequence were inserted at the same site. The miR-22 expression plasmid was constructed by cloning into the pCDNA3 expression plasmid (Invitrogen) via BamHI and EcoRI sites a DNA fragment encoding the pre-miR-22 stem & loop that was amplified from genomic DNA by PCR. Expression plasmids for miR-22 and control antagomirs were constructed as previousely described [28] by inserting oligonucleotides complementary to miR-22, or a random sequence, into the pSUPER plasmid. The probes for the run-on experiments were first amplified from genomic DNA by PCR and then inserted into pTZ57R/T (Fermentas). All the constructs were verified by sequencing. The FoxO1 fused to GFP were generously provided by Prof. Terry Unterman (University of Illinois, Chicago) and the AKT-DN expression plasmid was provided by Rony Seger (Weizmann Institute).

### Transient transfection and primer extension assays

The cell lines used in this study were previously described [29], [30] and maintained in Dulbecco's modified Eagle's medium (DMEM) (Gibco-BRL) supplemented with 10% fetal calf serum (HyClone). Transfection into HEK293T cells was done as described [31]. 24 h after transfection cells were harvested and their luciferase and renilla activities were measured. For primer extension assays total RNA was prepared 24 hours after transfection using Tri-reagent (MRC inc.). Primer extension was performed as described previously [29] using 20 µg of total RNA. Results were visualized with a Phosphoimager (Fuji, BAS 2500).

For northern and western blotting and for the AKT kinetics experiments, HeLa, MCF-7, and NIH3T3 cells were transfected using ICAFectin®441 (Eurogentec) according to manufacturers instructions. Positive transfectants were selected 24 hours after transfection using puromycin for 24 more hours.

### Nuclear run-on assay

For the run-on procedure DNA probes were amplified by PCR from the corresponding plasmids. The DNA fragments were then denatured with 0.25 M NaOH and 0.5 M NaCl for 5 minutes at room temperature, and transferred to ice. 500 ng of each probe was loaded onto a GeneScreen-Plus membrane (NEN) and allowed to dry. The membrane was neutralized in 0.5 M NaCl and 0.5 M Tris [pH 7.5] for 1 minute at room temperature, and then in 2X SSC. The probes were then UV crosslinked to the membrane. Nuclei were isolated from 150 mm plates of HEK293T cells. Cells were washed with ice-cold phosphate-buffered saline, harvested, resuspended in lysis buffer (10 mM NaCl, 3 mM MgCl_2_, 10 mM Tris-Hcl [pH 7.4]) and incubated for 30 minutes at 4°C. Cell membranes were then mechanically broken using a 2 ml glass homogenizer, and nuclei were separated from the cell debris by centrifugation. Nuclei were then resuspended in 100 µl storage buffer (40% glycerol, 50 mM Tris [pH 8.5], 5 mM MgCl_2_, 0.1 mM EDTA). For the run-on transcription 50 µl nuclei were used for each reaction. The α-amanitin (20 µg/ml) was added 5 minutes prior the reaction. The nuclei were supplemented with 25 µl of 4X reaction mix (100 mM HEPES [pH 7.5], 10 mM MgCl_2_, 10 mM DTT, 300 mM KCl, 20% glycerol), 12.5 µl of 8X tri-phosphate mix (14 µl 25 mM ATP, 14 µl 25 mM GTP, 14 µl 25 mM CTP, 0.4 µl 1 mM UTP and 83 µl ddH_2_O), 5 µl [^32^P] UTP (500 µCi), and ddH_2_O to a final volume of 100 µl. After 20 minutes at room temperature 2 µl of DNase I (Promega) were added, and the nuclei were incubated for 10 minutes at 37°C. The reaction was then stopped by 100 µg tRNA, 300 µg proteinase K and 300 µl of stop buffer (2% SDS, 7 M urea, 0.35 M NaCl, 1 mM EDTA, 10 mM Tris [pH 8.0]), and incubated for 2 hours at 42°C. To precipitate RNA, 50 µl of ice cold TCA was added, and the tubes were incubated for 20 minutes at 4°C, centrifuged and washed with ice cold absolute ethanol. The RNA pellet was resuspended in 50 µl of TE containing 0.5% SDS, and dissolved by incubating at 65°C for 30 minutes. The resultant radiolabeled RNA was added to the membrane that was prehybridized in 2.5 ml of hybridization buffer (50% formamide, 6X SSC, 10X Denhardt's solution, 0.2% SDS) for 6 hours at 42°C. The radiolabeled RNA was incubated for 72 hours with the membrane at 42°C, and then washed once with 6X SSC, 0.2% SDS, twice with 2X SSC, 0.2% SDS and twice more with 0.2X SSC, 0.2% SDS. The membranes were visualized with a Phosphoimager (Fuji, BAS 2500).

### Small RNA Northern Blot

For northern blot assay, small RNAs were extracted from HEK293T or HeLa cells by first preparing total RNA with Tri-reagent (MRC inc.) according to the manufacturer's instructions and then precipitating out large RNA with 0.1% NaCl and 5% PEG-8000 and centrifuging at 12000 rpm for 10 minutes at 4°C. Small RNAs were precipitated out from the supernatant by ethanol. Small RNA (5 µg) was loaded onto a 15% acrylamide gel, and run at 180V for 90 minutes, in 1X TBE. RNA was then transferred from the gel to a GeneScreen-Plus membrane (NEN), at 200 mA for 2 h, in 0.5X TBE. Next, the RNA was crosslinked to the membrane with UV irradiation. Pre-hybridization was carried out at 42°C for 2 h in hybridization buffer (5X SSC, 20 mM Na_2_HPO_4_ (pH 7.2), 7% SDS, and 2X Denhardt's solution), after which the ^32^P labeled probe was added. Hybridization was carried out 16–24 hours at 42°C. The membrane was then washed 3 times at 42°C in a washing solution (3X SSC, 25 mM NaH_2_PO_4_ (pH 7.5), 5% SDS, and 10X Denhardt's solution). Hybridization products were visualized using phosphoimager (Fuji, BAS 2500).

### Western blot and antibodies

HeLa (tet off), HEK293T and MCF7 cells were harvested and lysed in reporter lysis buffer (Promega). Protein concentrations were determined by Bradford protein assay (Bio-Rad). Samples were subjected to SDS-PAGE and proteins were transferred to nitrocellulose membrane and subjected to standard western blot procedure. Antibodies against PTEN, Sp1 and p300 were from SantaCruz biotechnology and alpha-tubulin from Sigma. Proteins were detected using the enhanced chemiluminescence kit (Pierce) and bands quantified with Science Lab 2003 Image Gauge (Fujifilm, Tokyo, Japan).

### Microscopic analysis of GFP-FoxO1 sub-cellular localization

NIH3T3 cells were co-transfected with FoxO1-GFP (kindly provided by Prof. Terry Unterman) together with either miR-22 expression plasmid or the parental pCDNA3. After 24 hours, the transfected cells were split to keep them at a low density and the microscopic analyses were carried out 48 hours after transfection. Time-Laps microscopy was performed using Olympus IX71 microscope (Delta-Vision - Applied Precision). The transfected cells were grown in an 8-chamber Lab-Tek Chambered Coverglass (Nunc). 10 fields for each transfection in which GFP-FoxO1 could be observed in the cytoplasm were chosen for analysis before and after serum deprivation. Images were taken every two minutes for 30 minutes after starvation. For larger scale analysis, we took images of the transfected cells that were seeded in a 6-well plate, using Nikon Eclipse TiS microscope. We randomly took images of 15–20 fields (30–50 cells) at four different time points: before starvation (time zero), 15 and 90 minutes after replacing the cell medium with serum-free medium, and then15 minutes after replacing the medium of starved cells with 10% FBS containing medium (Re-Feed). Image analysis was carried out using Science-Lab's ImageGauge (Fujifilm) in the following manner: densitometry of every cell expressing FoxO1-GFP was measured both in the nucleus and in the cytoplasm in an identical sample measure area. After reducing the background the nucleus/cytoplasm ratio was calculated. Final analysis is the sum of three independent experiments. Statistical analysis of the data was carried out by two-way ANOVA using SPSS program.

### Analysis of phosphorylated AKT

MCF-7 cells were co-transfected in a 6-well dish with miR-22 expression plasmid, and a puromycine resistance plasmid, using ICAFectin, according to the manufacture instructions. Twenty-four hours post transfection, transfected cells were selected with puromycine (0.5 µg/ml) and 48 hours post transfection cells were starved for 7 hours, followed by 5 minutes serum stimulation. The cells were then put on ice, washed 3 times with ice-cold PBS and harvested in 150 µl ice-cold RIPA with fresh protease inhibitor cocktail (Sigma). The lysates were then mixed (vortex), incubated on ice for 10 minutes, mixed again, and incubated for additional 10 minutes on ice, followed by 15 minutes centrifugation at 4°C, at 14,000 rpm. Supernatant was used for a western blot.

## Supporting Information

Figure S1 Chromatin immunoprecipitation assay using antibodies against RNA pol I, II and III. Shown are PCR amplification of the promoter region of 5S rRNA, β-actin, tRNAtyr, and endogenous miR-22 genes. rRNA, β-actin and tRNAtyr are indicative of Pol I, Pol II and Pol III genes, respectively.
(0.06 MB TIF)

Figure S2 Luciferase gene under the control of miR-22 promoter was transfected into HEK293T cell together with expression plasmid of wild type or dominant negative mutant NRF1 or NRF1 RNAi or empty expression plasmid (pSG5) as indicated.
(0.08 MB TIF)

Figure S3 The upper panel shows a representative western blot analyses with PTEN, SIRT1, SP1, p300, and tubulin antibodies, of cell lystates prepared from HeLa transfected with miR-22 expression plasmid or the parental vector pCDNA3. Quantitative analysis by densitometry of 3 experiments is shown in the lower panel.
(0.12 MB TIF)

Figure S4 MCF-7 cells were transfected with miR-22 expression plasmid or the parental vector pCDNA3 and 48 hours later the cells were serum starved for 7 hours. Then serum was added for 5 minutes and cell lysates were prepared. The upper panel shows a representative western blot analysis with the indicated antibodies and the graph at the lower panel represents a densitometric analysis of two independent transfection experiments.
(0.09 MB TIF)

Figure S5 The effect of miR-22 expression on the sub-cellular localization of FoxO1 is AKT dependent. NIH3T3 cells were co-transfected with FoxO1-GFP together with either miR-22 expression plasmid or the parental pCDNA3 and with or without a dominant negative mutant of AKT (AKT-DN). 48 hours after transfection the cells were subjected to serum starvation followed by serum addition to the starved cells. Images were taken at four different time points: before starvation (time 0), 15 and 90 minutes after replacing the cell medium to serum-free medium, and 15 minutes after addition of serum to the starved cells (Re-Feed).
(0.38 MB TIF)
