# Supplementary figures and images for: miR-22 Forms a Regulatory Loop in PTEN/AKT Pathway and Modulates Signaling Kinetics
Source: PLoS One. 2010 May 27;5(5):e10859. doi: 10.1371/journal.pone.0010859 (PMC2877705; doi:10.1371/journal.pone.0010859)

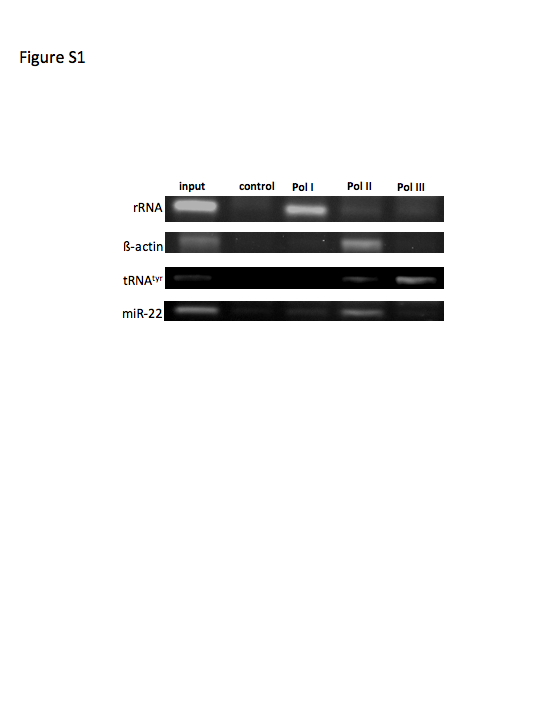

Supplement: Figure S1 — Chromatin immunoprecipitation assay using antibodies against RNA pol I, II and III. Shown are PCR amplification of the promoter region of 5S rRNA, β-actin, tRNAtyr, and endogenous miR-22 genes. rRNA, β-actin and tRNAtyr are indicative of Pol I, Pol II and Pol III genes, respectively. (0.06 MB TIF) [file pone.0010859.s001.tif]

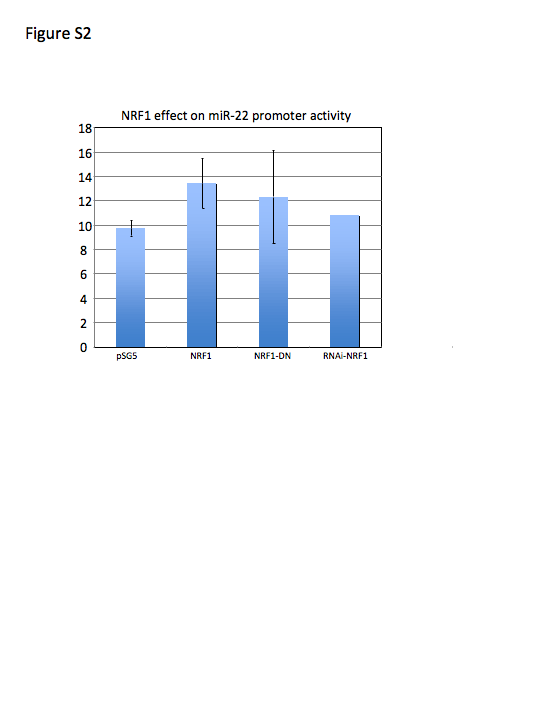

Supplement: Figure S2 — Luciferase gene under the control of miR-22 promoter was transfected into HEK293T cell together with expression plasmid of wild type or dominant negative mutant NRF1 or NRF1 RNAi or empty expression plasmid (pSG5) as indicated. (0.08 MB TIF) [file pone.0010859.s002.tif]

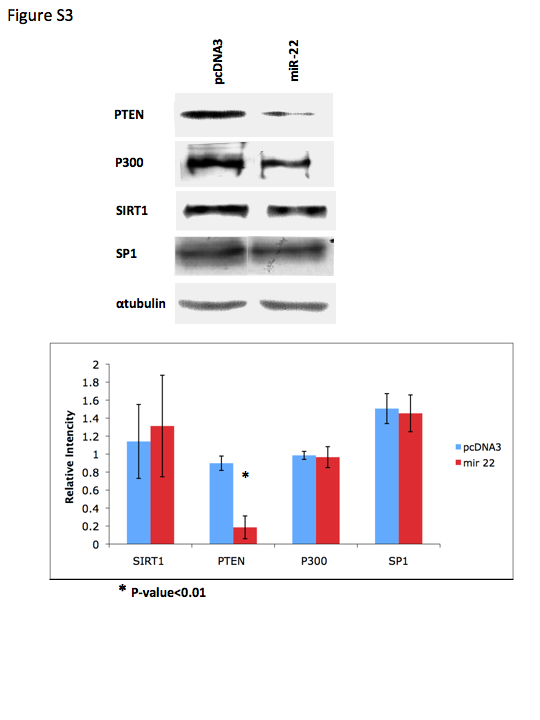

Supplement: Figure S3 — The upper panel shows a representative western blot analyses with PTEN, SIRT1, SP1, p300, and tubulin antibodies, of cell lystates prepared from HeLa transfected with miR-22 expression plasmid or the parental vector pCDNA3. Quantitative analysis by densitometry of 3 experiments is shown in the lower panel. (0.12 MB TIF) [file pone.0010859.s003.tif]

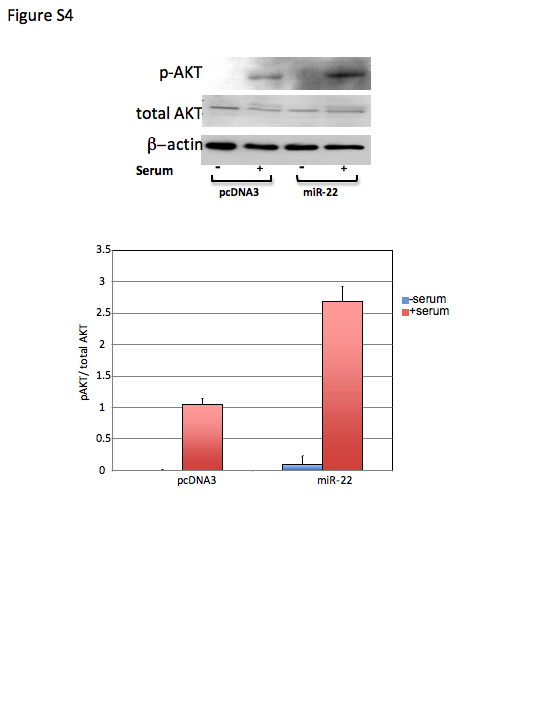

Supplement: Figure S4 — MCF-7 cells were transfected with miR-22 expression plasmid or the parental vector pCDNA3 and 48 hours later the cells were serum starved for 7 hours. Then serum was added for 5 minutes and cell lysates were prepared. The upper panel shows a representative western blot analysis with the indicated antibodies and the graph at the lower panel represents a densitometric analysis of two independent transfection experiments. (0.09 MB TIF) [file pone.0010859.s004.tif]

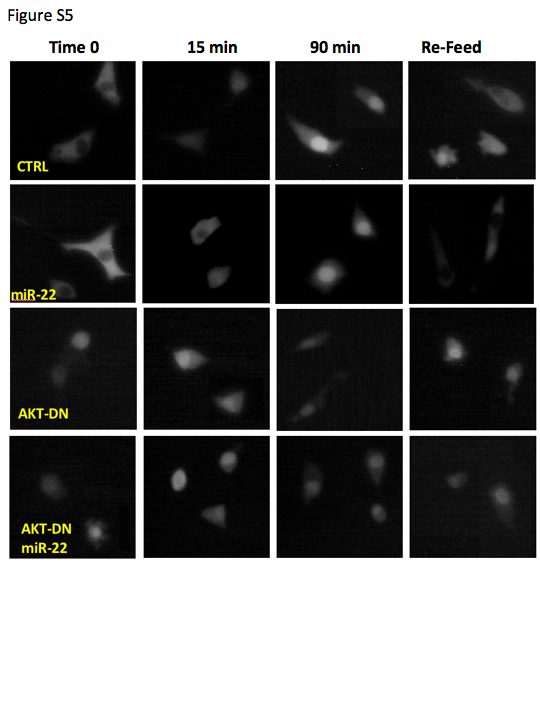

Supplement: Figure S5 — The effect of miR-22 expression on the sub-cellular localization of FoxO1 is AKT dependent. NIH3T3 cells were co-transfected with FoxO1-GFP together with either miR-22 expression plasmid or the parental pCDNA3 and with or without a dominant negative mutant of AKT (AKT-DN). 48 hours after transfection the cells were subjected to serum starvation followed by serum addition to the starved cells. Images were taken at four different time points: before starvation (time 0), 15 and 90 minutes after replacing the cell medium to serum-free medium, and 15 minutes after addition of serum to the starved cells (Re-Feed). (0.38 MB TIF) [file pone.0010859.s005.tif]
